# Echocardiographic Assessment of Structural and Functional Cardiac Alterations in Gestational Hypertension and Preeclampsia: A Comparative Cross-Sectional Study

**DOI:** 10.7759/cureus.92324

**Published:** 2025-09-14

**Authors:** Ramya Swaminathan, Priya Sharon

**Affiliations:** 1 Obstetrics and Gynaecology, Government Medical College and Hospital, Ariyalur, Ariyalur, IND; 2 Obstetrics and Gynaecology, Government Kilpauk Medical College, Chennai, IND

**Keywords:** atrial function, cardiovascular, cross-sectional studies, echocardiography, hypertension, pregnancy complications, pregnancy-induced, ventricular dysfunction, ventricular remodeling

## Abstract

Background

Hypertensive disorders of pregnancy (HDP), encompassing gestational hypertension and preeclampsia, are significant cardiovascular complications and leading causes of maternal morbidity and mortality. These conditions involve complex pathophysiological mechanisms extending beyond elevated blood pressure and induce cardiac structural and functional adaptations with potential long-term cardiovascular implications. Contemporary evidence demonstrates that women with hypertensive pregnancy disorders face substantially elevated risks for future cardiovascular disease. This comparative cross-sectional investigation aimed to systematically evaluate echocardiographic differences between pregnant women with gestational hypertension/preeclampsia and normotensive controls, providing a detailed characterization of cardiac structural and functional parameters to inform obstetric practice.

Methodology

This cross-sectional study was conducted at the Department of Obstetrics and Gynecology, Government Kilpauk Medical College, Chennai, following institutional ethics approval (IEC.NO-317/2020) with written informed consent. The investigation included 80 pregnant women aged 18-34 years in the third trimester, equally allocated into hypertensive (n=40) and normotensive control (n=40) groups through systematic age and parity matching. Inclusion criteria encompassed pregnant patients with systolic blood pressure ≥140 mmHg and diastolic blood pressure ≥90 mmHg developing after 20 weeks of gestation. Comprehensive echocardiographic assessment was performed using standardized protocols, evaluating left ventricular morphology, systolic function, diastolic performance, left atrial volumetric measurements, and pulmonary hemodynamics.

Results

Demographic characteristics demonstrated successful matching between groups (age: 25.55 vs 25.88 years, p=0.753; parity distribution: p=0.263). Proteinuria assessment showed 85% of hypertensive patients versus 5% of controls with a protein-to-creatinine ratio ≥0.3 (p<0.001). Left ventricular structural analysis revealed significant chamber dilatation in hypertensive patients (left ventricular end-diastolic diameter (LVEDD): 50.75 vs 41.60 mm, p<0.001; left ventricular end-systolic diameter (LVESD): 34.48 vs 26.83 mm, p<0.001) with adaptive wall thickening (left ventricular posterior wall systolic (LVPWs): 1.16 vs 0.95 mm, p<0.001). Diastolic function assessment demonstrated impaired relaxation patterns, including a reduced E/A ratio (1.31 vs 1.51, p=0.010), elevated E/e′ ratio (9.05 vs 7.63, p<0.001), and prolonged mitral valve deceleration time (144.50 vs 123.35 ms, p<0.001). Left atrial volumetric analysis revealed substantial enlargement (left atrial volume index (LAVI): 31.52 vs 17.56 mL/m², p<0.001). Pulmonary artery systolic pressure was significantly elevated (18.70 vs 16.93 mmHg, p<0.001), while systolic function remained preserved (ejection fraction: 67.23% vs 65.15%, p=0.311).

Conclusion

This echocardiographic investigation demonstrates significant cardiac structural and functional alterations in gestational hypertension and preeclampsia, characterized by ventricular remodeling, diastolic dysfunction, and pronounced left atrial enlargement. These findings highlight cardiac adaptations in hypertensive pregnancy disorders that warrant further multicenter investigation to determine clinical applications for screening and risk assessment.

## Introduction

Hypertensive disorders of pregnancy (HDP), encompassing gestational hypertension and preeclampsia, represent a significant global health challenge, complicating 2-8% of pregnancies worldwide and constituting a leading cause of maternal and perinatal morbidity and mortality [1,2]. The pathophysiological complexity of these conditions extends beyond simple blood pressure elevation, involving profound cardiovascular adaptations that may have lasting implications for maternal health [3,4]. Contemporary evidence demonstrates that women with HDP face substantially elevated risks for future cardiovascular disease, with studies indicating up to a fourfold increased risk of subsequent cardiovascular events [5,6].

The cardiovascular system undergoes remarkable physiological adaptations during normal pregnancy, including increased cardiac output, plasma volume expansion, and reduced systemic vascular resistance [7,8]. However, in pregnancies complicated by hypertensive disorders, these adaptive mechanisms become dysregulated, resulting in pathological cardiac remodeling characterized by increased afterload, altered preload conditions, and compromised ventricular function [2,9]. Dysregulation of the renin-angiotensin-aldosterone system observed in preeclampsia contributes to vasoconstriction, sodium retention, and cardiac structural changes that distinguish these conditions from normotensive pregnancy adaptations [10,11].

Echocardiographic assessment has emerged as a valuable noninvasive diagnostic modality for evaluating cardiac structure and function in HDP, providing critical insights into disease severity and progression [12,13]. Systematic reviews have identified increased vascular resistance and left ventricular mass as the most consistent echocardiographic findings in gestational hypertension and preeclampsia, with differentiating features including left ventricular wall thickness ≥ 1.0 cm and an exaggerated reduction in early-to-late diastolic filling velocity ratios [2]. Advanced echocardiographic techniques, including tissue Doppler imaging and speckle-tracking analysis, have revealed subtle but significant alterations in myocardial mechanics even in subclinical disease stages [14-16].

Recent investigations have demonstrated that cardiac dysfunction in HDP extends beyond the peripartum period, with persistent structural and functional abnormalities detectable months to years after delivery [17,18]. Left ventricular hypertrophy, diastolic dysfunction, and atrial remodeling are observed in association with disease severity and clinical outcomes [19,20]. Furthermore, these cardiac alterations appear to serve as early markers of future cardiovascular risk, with implications for long-term maternal health surveillance and intervention strategies [21,22].

The heterogeneity of hypertensive pregnancy disorders necessitates comprehensive echocardiographic characterization to optimize risk stratification and clinical management approaches [1,23]. Contemporary evidence suggests that early detection of cardiac dysfunction through systematic echocardiographic screening may facilitate timely interventions and improve both immediate and long-term maternal outcomes [24,25]. However, standardized protocols for cardiac assessment in HDP remain limited, highlighting the need for systematic comparative studies to establish evidence-based guidelines for echocardiographic evaluation in this high-risk population.

This comparative cross-sectional study aims to comprehensively evaluate echocardiographic differences between pregnant women with gestational hypertension/preeclampsia and normotensive controls, providing detailed characterization of cardiac structural and functional parameters that may inform clinical decision-making and risk assessment strategies in contemporary obstetric practice.

## Materials and methods

Study design and study setting

This investigation employed a comparative cross-sectional study design to evaluate echocardiographic differences between pregnant women with gestational hypertension/preeclampsia versus normotensive controls. Echocardiographic assessment was performed once during the third trimester (28-40 weeks’ gestation) for each participant. The study was conducted at the Department of Obstetrics and Gynecology, Government Kilpauk Medical College, Chennai, India, a tertiary care referral center providing comprehensive maternal-fetal healthcare services to a diverse urban and semi-urban population.

Study period

The research was conducted over 18 months, allowing adequate time for patient recruitment, comprehensive echocardiographic assessment, and systematic data collection procedures, while ensuring consistent methodological protocols throughout the investigation period.

Ethics committee approval

Prior to study initiation, comprehensive ethical approval was obtained from the Institutional Ethics Committee of Government Kilpauk Medical College, Chennai, ensuring adherence to the Declaration of Helsinki principles and Good Clinical Practice guidelines (IEC.NO-317/2020). Written informed consent was obtained from all participants following a detailed explanation of study procedures, potential risks, and benefits. Confidentiality protocols were strictly maintained, with participant anonymity preserved through coded identification systems.

Inclusion criteria

Pregnant patients aged 18-34 years with systolic blood pressure ≥140 mmHg and diastolic blood pressure ≥90 mmHg developing after 20 weeks of gestation, confirmed on repeated examinations at least 6 hours apart, were included. Participants were subsequently classified as having gestational hypertension (without proteinuria) or preeclampsia (with proteinuria defined as trace to 2+ on dipstick or spot protein-to-creatinine ratio >0.3). Pregnant women with gestational age 28-40 weeks, as calculated from the last menstrual period and dating scan, and with no previous history of essential hypertension were eligible for enrollment. Age- and parity-matched normotensive pregnant patients attending the outpatient department or admitted under similar gestational-age criteria served as controls.

Exclusion criteria

Patients with a prior history of hypertension, recurrent gestational hypertension, multiple gestation, or medical disorders of pregnancy, including valvular or congenital heart disease, cardiomyopathy, pulmonary hypertension, prior cardiac surgery, pulmonary embolism, systemic lupus erythematosus, connective tissue disease, antiphospholipid syndrome, interstitial lung disease, or chronic obstructive pulmonary disease, were excluded. Additionally, patients with anemia complicating pregnancy were excluded to eliminate potential confounding cardiovascular effects.

Sample size estimation

Sample size calculation was based on the study by Shivananjiah C et al. (2016) [26], which investigated echocardiographic changes in hypertensive disorders of pregnancy (n=24), considering the mean and SD of left ventricular systolic mass as 87.1 ± 4.65 in the hypertensive group and 84 ± 3.86 in the normotensive group, at a 95% confidence interval and 90% power. Using the formula,

$N = \frac{(Z_{1-\alpha/2} + Z_{1-\beta})^2 \times 2 \times \sigma^2}{(\mu_1 - \mu_2)^2}$

where Z₁₋ₐ/₂ = 1.96 for 95% confidence interval, Z₁₋β = 1.28 for 90% power, μ₁ = 87.1, μ₂ = 84, and σ = 4.26 (average SD), the calculated sample size was N = (1.96 + 1.28)² × 2 × 4.255²/(87.1 - 84)² = 39.59. Therefore, the required sample size for each group was 40, yielding a total sample size of 80 participants.

Sampling method

A convenience sampling methodology was employed for patient recruitment, with consecutive eligible patients presenting to the obstetrics outpatient department or admitted to the maternity ward during the study period systematically approached for participation following informed consent procedures.

Data collection procedure

Each participant underwent a comprehensive clinical history, including detailed obstetric history, medical comorbidities, and medication use. Physical examination encompassed anthropometric measurements, including weight, height, body mass index calculation, and body surface area estimation. Blood pressure was measured using a conventional sphygmomanometer with patients in the sitting position and the arm at heart level, with systolic and diastolic pressures determined using Korotkoff sounds. A comprehensive echocardiographic assessment was performed for all participants, evaluating left ventricular end-diastolic diameter (LVEDD), left ventricular end-systolic diameter (LVESD), left ventricular posterior wall (LVPW) systolic and diastolic thickness, ejection fraction, fractional shortening, early-to-late diastolic filling velocity ratio, tissue Doppler e′ velocity ratio, mitral valve deceleration time, left atrial volume and volume index (LAVI), pulmonary vein systolic diameter, pulmonary artery systolic pressure, and advanced parameters including Tei index measurements. Urine spot protein-to-creatinine ratio was measured systematically in both groups for diagnostic classification and severity assessment. All echocardiographic examinations were performed by trained personnel using standardized protocols. Due to clinical requirements, echocardiographers were aware of patient hypertensive status, representing a potential source of observer bias acknowledged in our limitations.

Data analysis

We entered the gathered data into Microsoft Excel (Microsoft Corporation, Redmond, WA) and analyzed it using SPSS version 26 (trial version; IBM Corp., Armonk, NY). Continuous variables were presented as mean ± SD and compared using independent-samples t tests, while categorical variables were expressed as frequencies and percentages with chi-square tests. Effect sizes were calculated using Cohen’s d, with interpretation thresholds of small (0.2), medium (0.5), and large (0.8). Statistical significance was defined as p < 0.05, with 95% confidence intervals calculated for all primary outcome measures to ensure comprehensive statistical interpretation and assessment of clinical relevance.

## Results

Table 1 presents the demographic and anthropometric characteristics of the study population. Both groups demonstrated comparable baseline characteristics, including age (25.55 ± 4.84 vs 25.88 ± 4.35 years, p = 0.753), weight (66.20 ± 12.92 vs 65.98 ± 12.73 kg, p = 0.953), BMI (29.18 ± 5.56 vs 27.25 ± 3.60 kg/m², p = 0.070), and body surface area (1.60 ± 0.15 vs 1.61 ± 0.14 m², p = 0.756). Parity distribution showed no significant difference between groups (primigravida: 55.0% vs 42.5%, p = 0.263). Proteinuria assessment revealed that 85% of hypertensive patients had a spot protein-to-creatinine ratio ≥ 0.3 compared to 5% of controls (p < 0.001), confirming appropriate diagnostic classification of the study groups.

**Table 1:** Demographic and anthropometric characteristics of study participants. Data are presented as mean ± standard deviation, except for parity status, which is presented as frequency (percentage). Statistical tests: Independent-samples t test for continuous variables; chi-square test for categorical variables. BSA: Body surface area.

Parameter	Hypertensive group (n = 40)	Normotensive group (n = 40)	Test statistic	p-value
Age (years)	25.55 ± 4.84	25.88 ± 4.35	t = -0.32	0.753
Weight (kg)	66.20 ± 12.92	65.98 ± 12.73	t = 0.08	0.953
Height (cm)	150.68 ± 6.81	153.03 ± 7.01	t = -1.53	0.06
BMI (kg/m²)	29.18 ± 5.56	27.25 ± 3.60	t = 1.84	0.07
BSA (m²)	1.60 ± 0.15	1.61 ± 0.14	t = -0.312	0.756
Parity status				
Primigravida	22 (55.0%)	17 (42.5%)	χ² = 1.25	0.263
Multigravida	18 (45.0%)	23 (57.5%)	-	-

Table 2 reveals significant structural alterations in left ventricular morphology among hypertensive patients. The markedly increased LVEDD (50.75 vs 41.60 mm, p < 0.001) and LVESD (34.48 vs 26.83 mm, p < 0.001) demonstrate significant ventricular chamber enlargement in hypertensive patients. Increased systolic wall thickness (LVPWs: 1.16 vs 0.95 mm, p < 0.001) was also observed. Remarkably, despite these structural changes, systolic function parameters (ejection fraction and fractional shortening) remained preserved, indicating compensated cardiac adaptation during early stages of hypertensive pregnancy disorders.

**Table 2:** Left ventricular morphology and systolic function parameters. Data are presented as mean ± SD. ^*^Statistically significant at p < 0.05. LVEDD: Left ventricular end-diastolic diameter; LVESD: Left ventricular end-systolic diameter; LVPWs: Left ventricular posterior wall systolic thickness; LVPWd: Left ventricular posterior wall diastolic thickness.

Parameter	Hypertensive group (n = 40)	Normotensive group (n = 40)	Independent-samples t test	p-value
LVEDD (mm)	50.75 ± 6.80	41.60 ± 3.43	t = 7.52	<0.001*
LVESD (mm)	34.48 ± 10.22	26.83 ± 2.54	t = 4.57	<0.001*
LVPWs (mm)	1.16 ± 0.17	0.95 ± 0.06	t = 7.42	<0.001*
LVPWd (mm)	0.83 ± 0.15	0.94 ± 0.11	t = -3.70	0.001*
Ejection fraction (%)	67.23 ± 6.77	65.15 ± 10.96	t = 1.02	0.311
Fractional shortening (%)	35.05 ± 3.78	34.15 ± 2.34	t = 1.13	0.26

Table 3 presents a comprehensive diastolic function assessment, revealing significant impairment in ventricular relaxation patterns among hypertensive patients. Diastolic function parameters showed a reduced E/A ratio (1.31 vs 1.51, p = 0.010), prolonged mitral valve deceleration time (144.50 vs 123.35 ms, p < 0.001), and an elevated E/e′ ratio (9.05 vs 7.63, p < 0.001). Pulmonary artery systolic pressure was higher in hypertensive patients (18.70 vs 16.93 mmHg, p < 0.001).

**Table 3:** Diastolic function assessment and pulmonary hemodynamics. Data are presented as mean ± SD. ^*^Statistically significant at p < 0.05. E/A: Early-to-late diastolic filling velocity ratio; E/e′: Mitral inflow E velocity to tissue Doppler e′ velocity ratio; MVDT: Mitral valve deceleration time; PVSD: Pulmonary vein systolic diameter; PASP: Pulmonary artery systolic pressure.

Parameter	Hypertensive group (n = 40)	Normotensive group (n = 40)	Independent-samples t test	p-value
E/A ratio	1.31 ± 0.43	1.51 ± 0.27	t = -2.44	0.010*
E/e′ ratio	9.05 ± 1.26	7.63 ± 1.10	t = 5.37	<0.001*
MVDT (ms)	144.50 ± 10.99	123.35 ± 10.56	t = 8.76	<0.001*
PVSD	1.04 ± 0.15	1.27 ± 0.11	t = -7.72	<0.001*
PASP (mmHg)	18.70 ± 1.81	16.93 ± 1.62	t = 4.60	<0.001*

Table 4 demonstrates significant left atrial chamber enlargement in hypertensive patients, with both absolute LA volume (34.58 vs 29.24 mL, p < 0.001) and indexed volume (31.52 vs 17.56 mL/m², p < 0.001) markedly increased. The reduced early diastolic time interval (Emsec: 85.35 vs 97.75 ms, p = 0.001) further corroborates impaired ventricular relaxation, while the preserved atrial contraction time (Amsec, p = 0.227) suggests maintained atrial systolic function. These findings collectively indicate that left atrial enlargement serves as a sensitive marker of diastolic dysfunction severity in gestational hypertensive disorders.

**Table 4:** Left atrial function and volumetric analysis. Data are presented as mean ± SD. ^*^Statistically significant at p < 0.05. LA: left atrium; LAVI: left atrial volume index; Emsec: early diastolic time interval; Amsec: atrial contraction time interval.

Parameter	Hypertensive group (n = 40)	Normotensive group (n = 40)	Independent-samples t test	p-value
LA volume (mL)	34.58 ± 1.65	29.24 ± 2.78	t = 10.40	<0.001*
LAVI (mL/m²)	31.52 ± 2.76	17.56 ± 2.33	t = 24.42	<0.001*
Emsec (ms)	85.35 ± 17.03	97.75 ± 16.34	t = -3.33	0.001*
Amsec (ms)	72.60 ± 22.35	67.20 ± 16.97	t = 1.22	0.227

Table 5 establishes the clinical classification and severity stratification of the study population. The marked difference in proteinuria prevalence (85% vs 5% with PCR ≥ 0.3, p < 0.001) confirms the appropriate diagnostic categorization of participants, with the majority of hypertensive patients demonstrating significant proteinuria consistent with preeclampsia rather than isolated gestational hypertension. The comparable gestational-age distribution between groups (p = 0.704) ensures that observed echocardiographic differences are attributable to hypertensive pathophysiology rather than gestational age-related cardiac adaptations, strengthening the validity of the comparative analyses.

**Table 5:** Proteinuria assessment and hypertensive classification. Data are presented as frequencies, with percentages in brackets. ^*^Statistically significant at p < 0.05. PCR: Protein-to-creatinine ratio.

Parameter	Hypertensive group (n = 40)	Normotensive group (n = 40)	Chi-square (χ²)	p-value
Spot PCR categories				
<0.3	6 (15%)	38 (95%)	51.71	<0.001*
≥0.3	34 (85%)	2 (5%)		
Gestational age distribution				
28-32 weeks	9 (22.5%)	9 (22.5%)	0.603	0.704
32-34 weeks	11 (27.5%)	14 (35%)		
>34 weeks	20 (50%)	17 (42.5%)		

Table 6 presents effect size calculations (Cohen’s d) for primary echocardiographic parameters between hypertensive and normotensive groups. The left atrial volume index demonstrated the largest effect size (d = 5.47, 95% CI: 4.78-7.00), followed by mitral valve deceleration time (d = 1.96, 95% CI: 1.37-2.55) and left ventricular posterior wall systolic thickness (d = 1.89, 95% CI: 1.31-2.47). Left ventricular end-diastolic diameter (d = 1.68, 95% CI: 1.12-2.24), E/e′ ratio (d = 1.21, 95% CI: 0.75-1.67), and pulmonary artery systolic pressure (d = 1.02, 95% CI: 0.56-1.48) showed large effect sizes according to Cohen’s criteria (d > 0.8).

**Table 6:** Statistical summary of primary echocardiographic outcomes. LVEDD: Left ventricular end-diastolic diameter; LAVI: Left atrial volume index; E/e′: Tissue Doppler ratio; PASP: Pulmonary artery systolic pressure; LVPWs: Left ventricular posterior wall systolic thickness; MVDT: Mitral valve deceleration time.

Outcome measure	Effect size (Cohen’s d)	95% CI	Clinical significance
LVEDD	1.68	1.12-2.24	Large effect
LAVI	5.47	4.78-7.00	Very large effect
E/e′ ratio	1.21	0.75-1.67	Large effect
PASP	1.02	0.56-1.48	Large effect
LVPWs	1.89	1.31-2.47	Large effect
MVDT	1.96	1.37-2.55	Large effect

## Discussion

This comparative cross-sectional investigation identified significant echocardiographic differences in cardiac structural and functional parameters between women with gestational hypertension/preeclampsia and normotensive controls. Our findings suggest substantial ventricular remodeling, diastolic dysfunction, and atrial enlargement in hypertensive pregnancy disorders.

The pronounced left ventricular chamber dilatation observed in our hypertensive cohort (LVEDD: 50.75 vs 41.60 mm, p < 0.001) aligns with previous investigations by Simmons LA et al. (2002), who reported similar ventricular enlargement patterns in 45 preeclamptic patients [27]. However, our findings contrast with the study by Bamfo JE et al. (2008), which demonstrated preserved ventricular dimensions in a smaller cohort (n = 28) of preeclamptic women with intrauterine growth restriction [21]. This discrepancy may reflect heterogeneity in disease severity and gestational age at assessment, differences in imaging protocols, ethnic variations, or sample size limitations, as emphasized by Castleman JS et al. (2016) in their systematic review of 36 studies encompassing 745 women with gestational hypertension and 815 with preeclampsia [2].

The adaptive wall thickening documented in our study (LVPWs: 1.16 vs 0.95 mm, p < 0.001) corresponds with findings reported by Rafik Hamad R et al. (2009), who observed increased left ventricular mass indices in preeclamptic patients (n = 57) compared with controls [28]. Scantlebury DC et al. (2015) documented left ventricular hypertrophy in women with hypertensive pregnancy disorders (n = 100) during postpartum follow-up, findings that align with the structural remodeling observed in our cross-sectional assessment [18]. These morphological adaptations likely represent compensatory mechanisms responding to elevated afterload conditions characteristic of hypertensive pregnancy disorders, as described in the comprehensive review by Hibbard JU et al. (2004) [9].

Our diastolic function assessment revealed significant impairment in ventricular relaxation properties, with reduced E/A ratios (1.31 vs 1.51, p = 0.010) and prolonged mitral valve deceleration times (144.50 vs 123.35 ms, p < 0.001). These findings are consistent with investigations by Marcolan Quitete CM et al. (2015), who documented similar diastolic dysfunction patterns in 42 chronic hypertensive pregnant women [29]. The elevated E/e′ ratios observed in our hypertensive group (9.05 vs 7.63, p < 0.001) indicate increased left ventricular filling pressures, corroborating results from Jiang L et al. (2023), who employed multimodal echocardiographic assessment in preeclampsia subtypes (n = 120) [14]. Advanced tissue Doppler studies by Pan G et al. (2019) similarly demonstrated impaired myocardial relaxation in severe preeclampsia patients (n = 60), supporting our findings of intrinsic diastolic dysfunction [16].

The substantial left atrial enlargement documented in our study (LAVI: 31.52 vs 17.56 mL/m², p < 0.001) represents a novel finding with significant clinical implications. While Mattioli AV et al. (2012) previously reported atrial remodeling in pregnant hypertensive women (n = 40), our investigation observed more substantial volumetric differences. The large effect size (d = 5.47) warrants careful interpretation and may reflect methodological variations, differences in measurement techniques, or characteristics specific to our study population [20]. The observed atrial enlargement may be associated with altered hemodynamics in hypertensive pregnancy, though cross-sectional data cannot establish whether this represents adaptation, pathology, or both. The preserved atrial contraction function (Amsec, p = 0.227) suggests a compensated rather than decompensated cardiac status, indicating early-stage pathological remodeling.

Our pulmonary hemodynamic assessment revealed modestly elevated pulmonary artery systolic pressures in the hypertensive group (18.70 vs 16.93 mmHg, p < 0.001), though both values remain within normal physiological ranges for pregnancy. While Çağlar FN et al. (2016) documented right heart dysfunction in preeclampsia (n = 45) using comprehensive right ventricular indices including TAPSE and strain imaging [30], our study focused exclusively on left-sided cardiac parameters and pulmonary pressures. Therefore, we cannot draw conclusions regarding biventricular involvement. The modest PASP elevation observed may reflect left atrial pressure transmission rather than primary pulmonary vascular pathology, though this interpretation remains speculative without direct right ventricular assessment.

The preserved systolic function parameters observed in our study (ejection fraction: 67.23% vs 65.15%, p = 0.311) align with investigations by Tatapudi and Pasumarthy (2017), who demonstrated maintained left ventricular systolic performance across the gestational hypertension severity spectrum (n = 120) [31]. However, these findings contrast with studies employing advanced imaging techniques, such as Mostafavi A et al. (2019), who detected subtle systolic dysfunction using speckle-tracking echocardiography in preeclamptic patients (n = 30) [15]. This discrepancy highlights the superior sensitivity of strain imaging compared with conventional ejection fraction measurements in detecting early myocardial dysfunction.

The proteinuria assessment in our investigation (85% vs 5% with PCR ≥ 0.3, p < 0.001) confirms appropriate diagnostic classification and disease severity stratification. Jain N et al. (2016) similarly emphasized the importance of proteinuria quantification in correlating echocardiographic findings with preeclampsia severity (n = 100) [32]. The high prevalence of significant proteinuria in our hypertensive cohort confirms that most participants had preeclampsia rather than isolated gestational hypertension, providing appropriate disease severity characterization for interpreting the observed cardiac alterations.

Our demographic matching between study groups helped minimize certain confounding variables that could influence echocardiographic parameters, though residual confounding from unmeasured factors remains possible given our convenience sampling approach. The comparable gestational-age distributions ensure that observed differences reflect pathological rather than physiological adaptations, as emphasized by Lan L et al. (2025) in their comprehensive review of maternal cardiac changes during pregnancy [8]. The comparable body mass index between groups (p = 0.070) further supports the attribution of cardiac alterations to hypertensive pathophysiology rather than metabolic factors.

Clinical significance

The echocardiographic alterations observed in our cross-sectional investigation may have clinical relevance, though longitudinal studies are required to determine their persistence and prognostic significance beyond the immediate peripartum period. The documented cardiac structural and functional alterations provide objective evidence supporting the implementation of systematic echocardiographic screening protocols in the management of gestational hypertension and preeclampsia, as advocated by Briller (2022) in recent expert recommendations [12].

The substantial left atrial enlargement observed in our hypertensive cohort represents a notable echocardiographic finding. While atrial dilatation has been associated with diastolic dysfunction severity in nonpregnant populations, the prognostic significance of this finding specifically in pregnancy requires dedicated longitudinal investigation. Curtis SL et al. (2023), in their position statement on behalf of the British Society of Echocardiography, emphasized the prognostic value of atrial volumetric assessment in pregnancy-related cardiac evaluation [13]. Our findings suggest that left atrial volume index measurement should be incorporated into routine echocardiographic assessment protocols for hypertensive pregnancy disorders.

The documented diastolic dysfunction patterns have immediate clinical relevance for peripartum fluid management and delivery planning strategies. Estensen ME et al. (2013) demonstrated that impaired ventricular relaxation properties persist into the postpartum period, potentially influencing anesthetic management and hemodynamic monitoring requirements during delivery [33]. The elevated filling pressures evidenced by increased E/e′ ratios in our study population warrant careful consideration of fluid balance optimization and may necessitate modified labor and delivery protocols to prevent cardiac decompensation.

Furthermore, our findings support emerging evidence regarding the long-term cardiovascular risk implications of hypertensive pregnancy disorders. Stuart JJ et al. (2018) demonstrated that women with preeclampsia face significantly elevated 10-year cardiovascular risk scores [5], while Arnott C et al. (2020) documented persistent cardiovascular risk elevation extending decades after delivery [3]. The cardiac structural alterations documented in our investigation provide mechanistic insights into these epidemiological observations, suggesting that pregnancy-related cardiac remodeling may represent an early manifestation of underlying cardiovascular susceptibility.

Our findings contribute to the growing body of evidence characterizing cardiac alterations in hypertensive pregnancy disorders. However, the development of standardized echocardiographic protocols requires multicenter validation studies and consensus guidelines. Current recommendations, such as those by Daubert MA et al. (2024), focus primarily on blood pressure monitoring rather than routine echocardiographic screening [34]. Our results suggest that echocardiographic parameters, particularly left atrial volume index and diastolic function measures, may serve as valuable biomarkers for risk stratification and long-term cardiovascular health prediction in this high-risk population.

Strengths

Our investigation demonstrates several methodological strengths that enhance the validity and clinical applicability of our findings. The systematic matching of study groups by age, parity, and gestational age reduces potential confounding variables that could influence echocardiographic parameters, though residual confounding from unmeasured factors cannot be excluded. The comprehensive echocardiographic assessment protocol encompasses multiple cardiac domains, structural parameters, systolic function, diastolic performance, and pulmonary hemodynamics, providing multidimensional cardiovascular evaluation beyond conventional measurements. The substantial effect sizes documented across primary outcome measures (Cohen’s d > 0.8) quantify the magnitude of observed differences between groups. Additionally, the standardized diagnostic criteria employed for hypertensive disorder classification, combined with objective proteinuria quantification, ensure appropriate patient categorization and enhance the comparability of our results with existing literature.

Limitations

Several methodological limitations warrant acknowledgment when interpreting our findings. The single-center design may limit the generalizability of our results to diverse populations with varying ethnic backgrounds, socioeconomic status, and healthcare delivery systems. The cross-sectional methodology precludes longitudinal assessment of cardiac function evolution throughout pregnancy and into the postpartum period, potentially missing dynamic changes in cardiovascular adaptation. The convenience sampling approach may introduce selection bias, as patients presenting to our tertiary care facility may represent more severe cases than community-based populations. Importantly, we did not perform a formal assessment of intra- and interobserver variability for echocardiographic measurements. Although standardized protocols were employed and measurements were performed by trained personnel, the absence of reproducibility metrics limits our ability to quantify measurement precision and may affect the reliability of our findings. This represents a significant methodological limitation that should be addressed in future investigations. The absence of advanced echocardiographic techniques, such as speckle-tracking strain analysis or three-dimensional volumetric assessment, may have limited our ability to detect subtle myocardial dysfunction not apparent on conventional imaging. Furthermore, the lack of long-term follow-up data prevents evaluation of the prognostic significance of the documented cardiac alterations for future cardiovascular outcomes. Finally, potential confounding factors, including medication effects, comorbid conditions, and lifestyle variables, were not comprehensively controlled in our analysis, which may have influenced the observed echocardiographic differences between study groups.

Recommendations

Future investigations should employ multicenter, longitudinal study designs to evaluate the temporal evolution of cardiac function throughout pregnancy and during extended postpartum follow-up. Advanced echocardiographic techniques, including speckle-tracking strain analysis and three-dimensional imaging, should be incorporated to enhance sensitivity for detecting subclinical myocardial dysfunction. Standardized protocols for echocardiographic assessment in hypertensive pregnancy disorders should be developed and validated across diverse clinical settings to optimize risk stratification and management strategies. Long-term cardiovascular outcome studies are essential to establish the prognostic significance of pregnancy-related cardiac alterations for future maternal health.

## Conclusions

This cross-sectional investigation identified significant cardiac structural and functional alterations in women with gestational hypertension and preeclampsia compared with normotensive controls. The observed changes, including ventricular chamber enlargement, increased wall thickness, diastolic dysfunction, and left atrial dilatation, suggest cardiac adaptations that extend beyond normal pregnancy-related physiological changes. While these findings contribute to understanding the cardiovascular pathophysiology of hypertensive pregnancy disorders, their prognostic significance requires validation through longitudinal studies. The documented alterations highlight the potential value of echocardiographic assessment in selected high-risk patients, though the development of screening protocols will require multicenter validation and consensus guidelines. Future research should focus on establishing the temporal persistence of these cardiac changes, their relationship to long-term cardiovascular outcomes, and the cost-effectiveness of systematic cardiac surveillance. These observations provide a foundation for further investigation into optimal risk-stratification strategies and may inform future approaches to cardiovascular monitoring in women with hypertensive pregnancy disorders.

## References

[1] Alhuneafat L, Alrifai N, Amoateng R (2024). Echocardiographic differences in women across subtypes of hypertensive disorders of pregnancy. JACC Adv.

[2] Castleman JS, Ganapathy R, Taki F, Lip GY, Steeds RP, Kotecha D (2016). Echocardiographic structure and function in hypertensive disorders of pregnancy: a systematic review. Circ Cardiovasc Imaging.

[3] Arnott C, Nelson M, Alfaro Ramirez M (2020). Maternal cardiovascular risk after hypertensive disorder of pregnancy. Heart.

[4] Groenhof TK, Zoet GA, Franx A, Gansevoort RT, Bots ML, Groen H, Lely AT (2019). Trajectory of cardiovascular risk factors after hypertensive disorders of pregnancy. Hypertension.

[5] Stuart JJ, Tanz LJ, Missmer SA, Rimm EB, Spiegelman D, James-Todd TM, Rich-Edwards JW (2018). Hypertensive disorders of pregnancy and maternal cardiovascular disease risk factor development: an observational cohort study. Ann Intern Med.

[6] Ying W, Catov JM, Ouyang P (2018). Hypertensive disorders of pregnancy and future maternal cardiovascular risk. J Am Heart Assoc.

[7] Cong J, Fan T, Yang X, Squires JW, Cheng G, Zhang L, Zhang Z (2015). Structural and functional changes in maternal left ventricle during pregnancy: a three-dimensional speckle-tracking echocardiography study. Cardiovasc Ultrasound.

[8] Lan L, Zhang C, Geng J, Li Q (2025). Echocardiographic assessment of the maternal heart: a review of the structural and functional changes during pregnancy. Echocardiography.

[9] Hibbard JU, Shroff SG, Lang RM (2004). Cardiovascular changes in preeclampsia. Semin Nephrol.

[10] Herse F, Dechend R, Harsem NK (2007). Dysregulation of the circulating and tissue-based renin-angiotensin system in preeclampsia. Hypertension.

[11] Verdonk K, Visser W, Van Den Meiracker AH, Danser AH (2014). The renin-angiotensin-aldosterone system in pre-eclampsia: the delicate balance between good and bad. Clin Sci (Lond).

[12] Briller JE (2022). Echocardiographic screening in hypertensive pregnancy disorders: probing the window of opportunity. J Am Coll Cardiol.

[13] Curtis SL, Belham M, Bennett S (2023). Transthoracic echocardiographic assessment of the heart in pregnancy-a position statement on behalf of the British Society of Echocardiography and the United Kingdom Maternal Cardiology Society. Echo Res Pract.

[14] Jiang L, Liang W, Xie R, Fang X, Liu D, Zhang M, Shi C (2023). Assessment of left ventricular structure and function in preeclampsia subtypes by multimodal echocardiography. J Obstet Gynaecol Res.

[15] Mostafavi A, Tase Zar Y, Nikdoust F, Tabatabaei SA (2019). Comparison of left ventricular systolic function by 2D speckle-tracking echocardiography between normal pregnant women and pregnant women with preeclampsia. J Cardiovasc Thorac Res.

[16] Pan G, Chen D, Xu L (2019). Cardiac dysfunction in women with severe preeclampsia detected by tissue Doppler and speckle-tracking echocardiography. Int J Clin Exp Med.

[17] Crowley R, Youssef G, Henry A (2022). Echocardiographic assessment of left ventricular structure and function in hypertensive disorders of pregnancy at six months and two years postpartum. Eur Heart J.

[18] Scantlebury DC, Kane GC, Wiste HJ (2015). Left ventricular hypertrophy after hypertensive pregnancy disorders. Heart.

[19] Kim MJ, Seo J, Cho KI, Yoon SJ, Choi JH, Shin MS (2016). Echocardiographic assessment of structural and hemodynamic changes in hypertension-related pregnancy. J Cardiovasc Ultrasound.

[20] Mattioli AV, Pennella S, Demaria F, Farinetti A (2012). Atrial remodeling in pregnant hypertensive women: comparison between chronic and gestational hypertension. Open Cardiovasc Med J.

[21] Bamfo JE, Kametas NA, Chambers JB, Nicolaides KH (2008). Maternal cardiac function in normotensive and pre-eclamptic intrauterine growth restriction. Ultrasound Obstet Gynecol.

[22] Haug EB, Horn J, Markovitz AR (2019). Association of conventional cardiovascular risk factors with cardiovascular disease after hypertensive disorders of pregnancy: analysis of the Nord-Trøndelag Health Study. JAMA Cardiol.

[23] Saraf A (2024). Echocardiographic changes in hypertensive disorders of pregnancy: just another finding or a clinical outcomes biomarker?. JACC Adv.

[24] Dennis AT, Castro JM (2014). Echocardiographic differences between preeclampsia and peripartum cardiomyopathy. Int J Obstet Anesth.

[25] Giorgione V, Di Fabrizio C, Giallongo E (2024). Angiogenic markers and maternal echocardiographic indices in women with hypertensive disorders of pregnancy. Ultrasound Obstet Gynecol.

[26] Shivananjiah C, Nayak A, Swarup A (2016). Echo Changes in Hypertensive Disorder of Pregnancy. J Cardiovasc Echogr.

[27] Simmons LA, Gillin AG, Jeremy RW (2002). Structural and functional changes in left ventricle during normotensive and preeclamptic pregnancy. Am J Physiol Heart Circ Physiol.

[28] Rafik Hamad R, Larsson A, Pernow J, Bremme K, Eriksson MJ (2009). Assessment of left ventricular structure and function in preeclampsia by echocardiography and cardiovascular biomarkers. J Hypertens.

[29] Marcolan Quitete CM, Marcolan Salvany A, de Andrade Martins W, Mesquita ET (2015). Left ventricular remodeling and diastolic function in chronic hypertensive pregnant women. Pregnancy Hypertens.

[30] Çağlar FN, Ozde C, Bostancı E (2016). Assessment of right heart function in preeclampsia by echocardiography. Pregnancy Hypertens.

[31] Tatapudi R, Pasumarthy LR (2017). Maternal cardiac function in gestational hypertension, mild and severe preeclampsia and normal pregnancy: a comparative study. Pregnancy Hypertens.

[32] Jain N, Verma A, Rajoria L (2016). Evaluation of echocardiographic systolic parameters in pre-eclamptics and normotensives women. J Obstet Gynaecol India.

[33] Estensen ME, Remme EW, Grindheim G, Smiseth OA, Segers P, Henriksen T, Aakhus S (2013). Increased arterial stiffness in pre-eclamptic pregnancy at term and early and late postpartum: a combined echocardiographic and tonometric study. Am J Hypertens.

[34] Daubert MA, Stebbins A, Peragallo-Urrutia R (2024). Early postpartum blood pressure screening is associated with increased detection of cardiovascular risk factors in women with hypertensive disorders of pregnancy. Am Heart J.

